# Supplementary material for: Intestinal Carriage of Two Distinct stx2f-Carrying Escherichia coli Strains by a Child with Uncomplicated Diarrhea
Source: Pathogens. 2024 Nov 15;13(11):1002. doi: 10.3390/pathogens13111002 (PMC11597368; doi:10.3390/pathogens13111002)
Supplement: Supplementary file 1 [file pathogens-13-01002-s001.zip › pathogens-3293178-supplementary-figures.pdf]

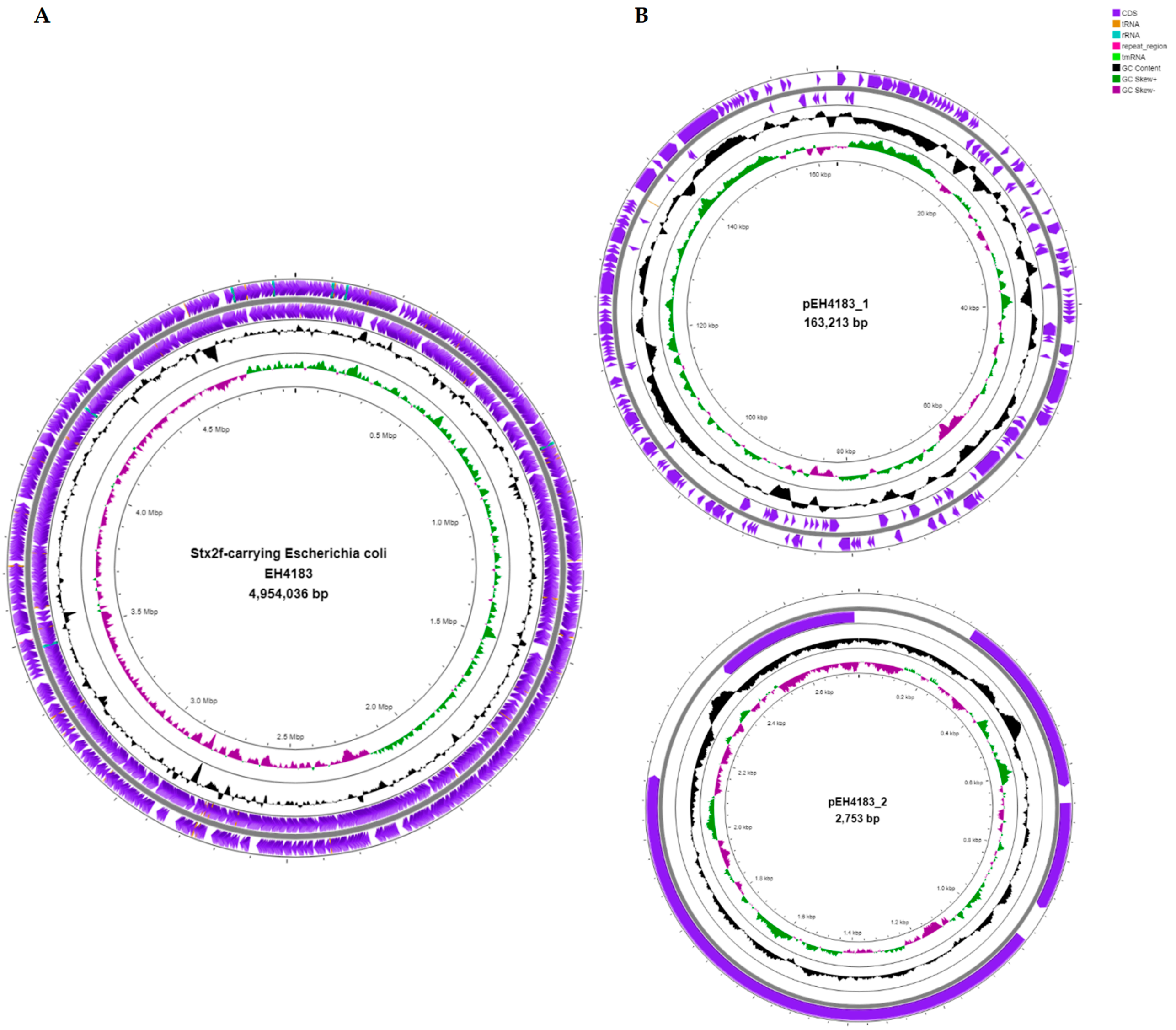

Figure S1. Circular maps of the chromosome (A) and two plasmids (B) of strain EH4183. From the outer circle moving inward, circles 1 and 2 represent protein-coding genes (CDS; purple), tRNA genes (tRNA; orange), and rRNA genes (rRNA; turquoise), repeat regions (repeat_region; pink) and transfer-messenger RNA (tmRNA; light green) on the positive and negative strands; circle 3 shows the GC content (CG Content; black); circle 4 shows the GC skew (positive GC skew [GC Skew+], green; negative GS skew [GC Skew-], purple; and circle 5 displays the genomic coordinates.

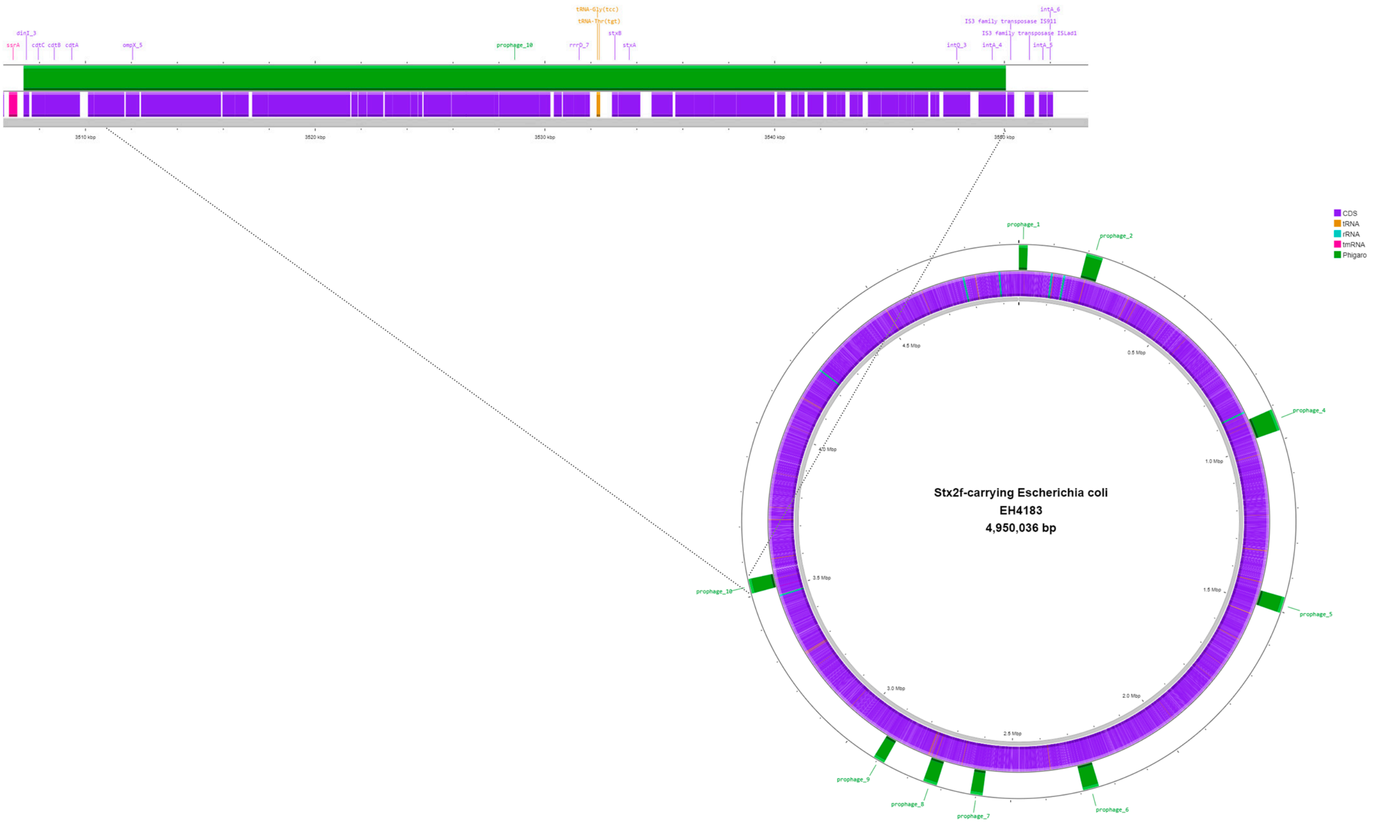

Figure S2. Circular map of the chromosome of STEC O63:H6 strain EH4183, with indication of the predicted prophages. The predicted prophages in the EH4183 genome using Phigaro are shown in dark green. A closer view on the *stx2f*-harboring prophage of 42.8 Kb and its surrounding region is given. Hypothetical proteins are not labelled on the prophage region of interest.

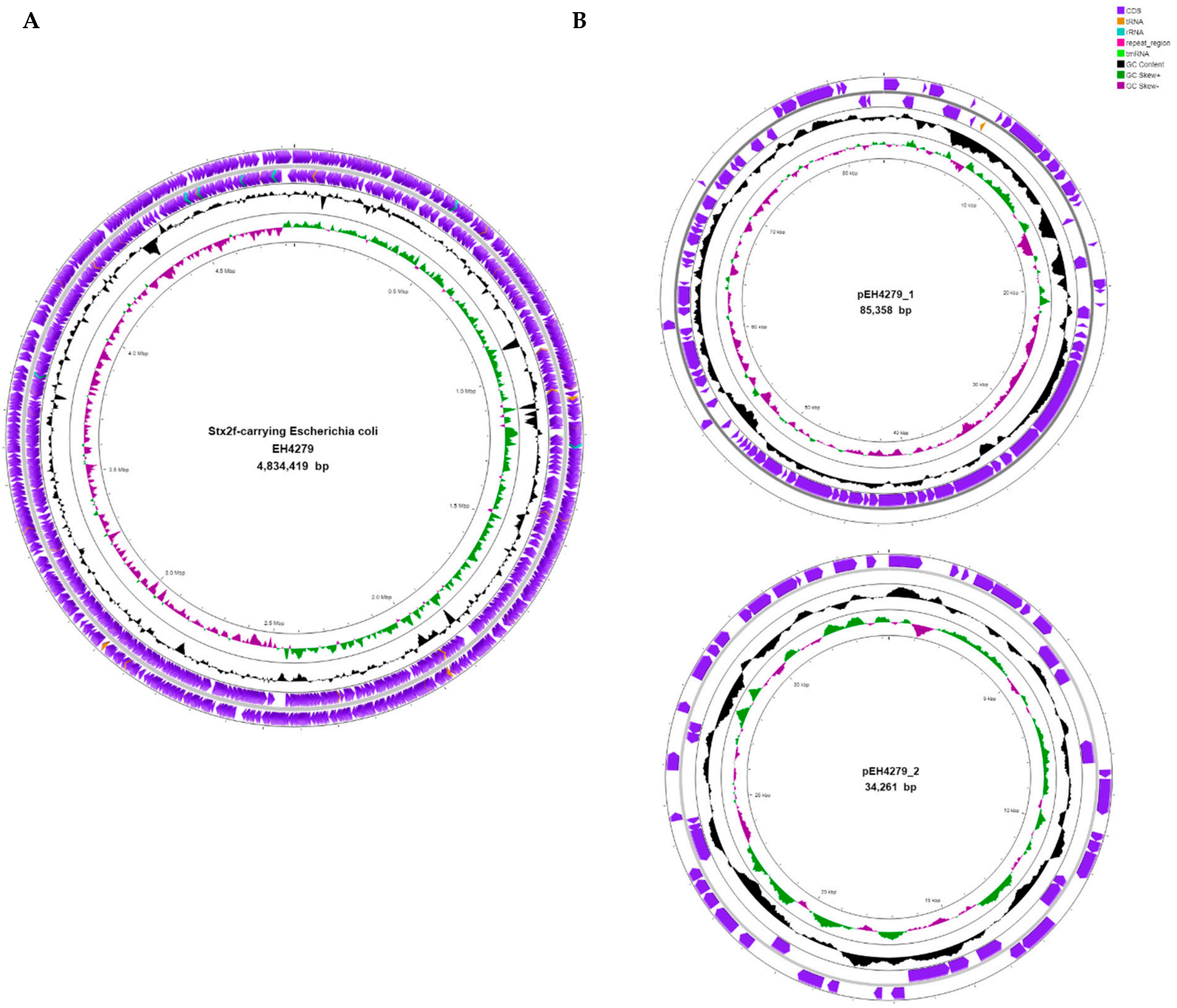

Figure S3. Circular maps of the chromosome (A) and two plasmids (B) of strain EH4279. From the outer circle moving inward, circles 1 and 2 represent protein-coding genes (CDS; purple), tRNA genes (tRNA; orange), and rRNA genes (rRNA; turquoise), repeat regions (repeat_region; pink) and transfer-messenger RNA (tmRNA; light green) on the positive and negative strands; circle 3 shows the GC content (CG Content; black); circle 4 shows the GC skew (positive GC skew [GC Skew+], green; negative GS skew [GC Skew-], purple; and circle 5 displays the genomic coordinates.

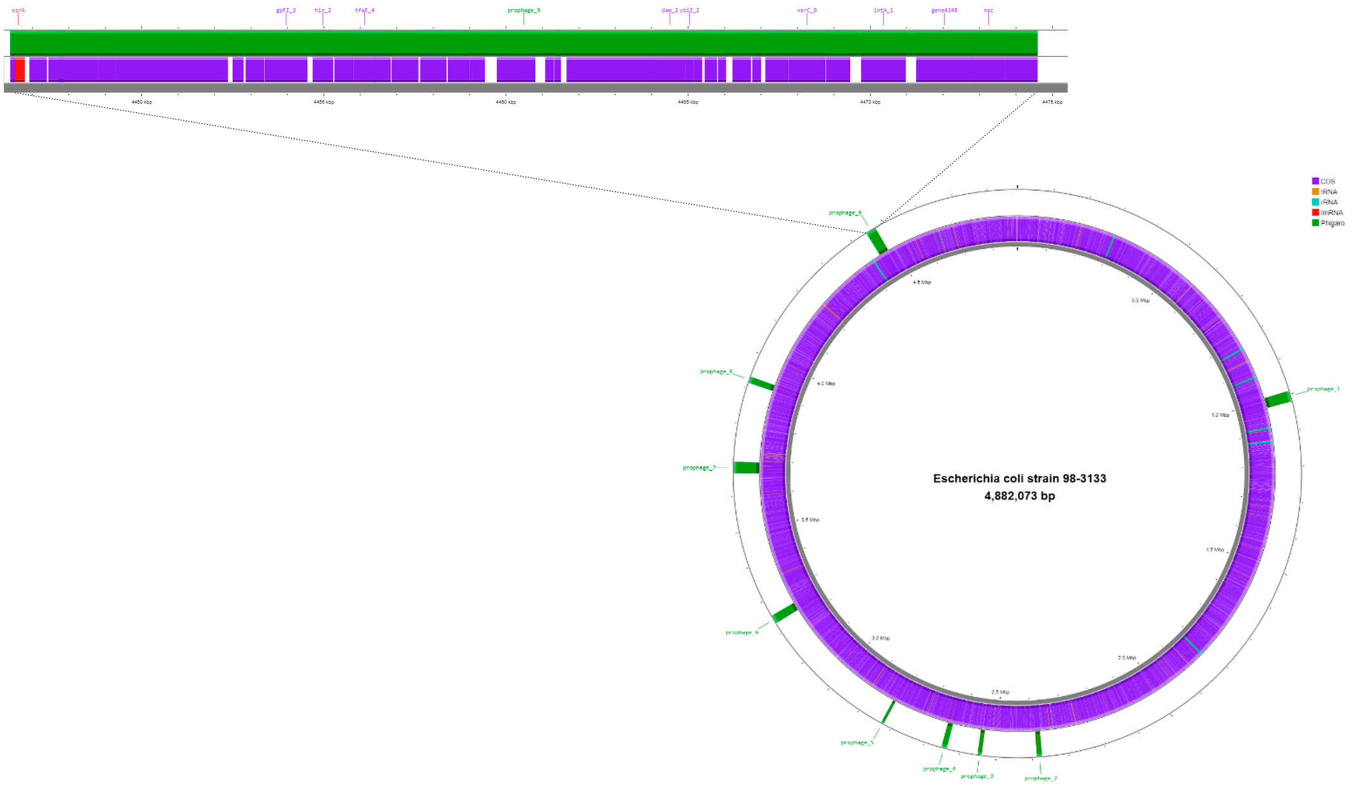

Figure S4. Circular map of the chromosome of *Escherichia coli* O157:H16 strain 98-3133, with indication of the predicted prophages. The predicted prophages in the 98-3133 genome using Phigaro are shown in dark green. A closer view on the *stx*-negative predicted prophage adjacent to the *ssrA* gene, which encodes a transfer-messenger RNA (tmRNA; red), is given. Hypothetical proteins are not labelled on the prophage region of interest.
