# Supplementary material for: Intestinal Carriage of Two Distinct stx2f-Carrying Escherichia coli Strains by a Child with Uncomplicated Diarrhea
Source: Pathogens. 2024 Nov 15;13(11):1002. doi: 10.3390/pathogens13111002 (PMC11597368; doi:10.3390/pathogens13111002)
Supplement: Supplementary file 1 [file pathogens-13-01002-s001.zip › Table S1_revised_v2.pdf]

**Table S1.** Non-exhaustive list of publications with characterized O157:H16 strains

| Country | Source | n | Pathotype | Achtman MLST | EcMLST | *eae* (subtype) [a] | *bfpA* [b] | *stx* | References |
|---|---|---|---|---|---|---|---|---|---|
| Argentina | Animal (dog) | 11 | aEPEC | NT | 171 | + (ε) | - | - | [23] |
| | Human | 4 | aEPEC | NT | NT | + (ε) | - | - | [30] |
| | Meat (chorizo) | 1 | NA | NT | NT | - | NT | - | [24] |
| China | Animal (duck) | 1 | NA | 1011 | NT | - | NT | - | [27,28] |
| | Animal (fish) | 1 | NA | 1011 | NT | - | NT | - | [28] |
| | Animal (chicken) | 1 | NA | 1011 | NT | - | NT | - | [28] |
| France | Meat | 2 | aEPEC | 10 | 171 | + (ε) | - | - | [22,23,25] |
| Germany | Human | 14 | aEPEC | NT | 171 | + (ε) | - | - | [21,23] |
| | Human | 2 | aEPEC | NT | 171 | + (β) | - | - | [23] |
| | Human | 4 | NA | NT | 344 | | NT | - | [23] |
| | Human | 1 | NA | NT | 83 | | NT | - | [23] |
| Ireland | Animal (cattle) | 1 | STEC | NT | NT | + | NT | *stx1 stx2* | [32] |
| Norway | Human | 2 | aEPEC | 10 | 171 | + (ε) | - | - | [20,23] |
| Sweden | Food (flour) | 1 | aEPEC | 10 | NT | + (ε) | - | - | [29] |
| The Netherlands | Environmental | 9 | aEPEC | NT | 171 | + (ε) | - | - | [23] |
| | Environmental | 1 | NA | NT | 344 | - | NT | - | [23] |
| | Human | 8 | aEPEC | 10 | NT | + (ε) | NT | - | [27] |
| USA | Animal (gazella slender horned) | 1 | aEPEC | 10 | NT | + (β) | - | - | [28] |
| | Animal (canine intestine) | 1 | aEPEC | 10 | NT | + (β) | - | - | [28] |
| | Human | 1 | aEPEC | 10 | NT | + (ε) | - | - | [27] |
| | Human | 2 | aEPEC | NT | 171 | + (β) | - | - | [22,23] |
| | Human | 1 | NA | NT | 344 | - | NT | - | [22,23] |
| | Human | 1 | NA | 5502 | 344 v1 | - | NT | - | [22,23,27] |
| | Meat | 1 | NA | NT | 344 | - | NT | - | [22,23] |
| | Meat | 1 | NA | NT | 344 v2 | - | NT | - | [22,23] |
| | Water | 6 | aEPEC | NT | 171 | + (ε) | - | - | [22,23] |

NT: not tested; [a] The intimin gene (*eae*) (subtype) highlighted in blue were not tested in the original papers. The prediction of the *eae* (subtype) was performed using VirulenceFinder 2.0 available from the Center for Genomic Epidemiology platform (https://www.genomicepidemiology.org/services/); [b] As mentioned in the original papers, strains without *eae* were not tested for *bfp*A.
